# Camphor an Antidote to Strychnia—Experiment—Failure, &c. &c.

**Published:** 1855-10

**Authors:** J. E. Thompson

**Affiliations:** Bates Co., Mo.


*Camphor an antidote to Strychnia—experiment—failure, &c. &c.*—Having read an article in the 51st vol. p. 476, of the Boston Medical and Surgical Journal, from Dr. Tewksbury, of Portland, Me., upon the antidotal effects of camphor upon the poison of strychnia, I have been induced to make an experiment with reference to it, and report the result thereof in your Journal. On the 1st of May, 1855, I procured two dogs of equal size, age, and health, for the purpose of experimenting. To the first I gave 1 grain of strychnia, followed immediately by the administration of 2 drachms of strong alcoholic tr. of camph.; in ten minutes, I gave another drachm. In twenty minutes from the time it took the poison, it fell, strongly convulsed, apparently dead; the fit lasted five minutes. When it recovered a little, I gave 2 drachms more of tr. camph.; in five minutes it had another fit, harder than the first, lasting ten minutes. It was scarcely recovered when it took another, and in five minutes died.

To the second, I gave ½ gr. strychnia, followed by 1 drachm of tr. camph. In ten minutes, I gave another drachm; and in ten minutes, another. In five minutes from the last dose of camphor, it had a light fit, which lasted but a few seconds. In ten minutes, I gave a drachm more, which was scarcely swallowed when it was taken with violent convulsions, and died in a few minutes.

In one hour after their death, I inspected the bodies. In both, serous effusions were present in the head, and its vessels filled with fluid blood. The lungs were in a highly congested state. The heart and its vessels contracted and empty. The bodies of both were flaccid, as soon as death took place. In the first, the stomach was much inflamed, of a deep violet tint, the poison adhering to its villous coat; detected, by the addition of nitric acid, when it assumed an orange-red, which soon passed to a golden yellow hue. The stomach of the second was but little affected; none of the poison could be detected on its coats. The intestines of both were tied into a perfect knot. The first died in about 45 minutes from the time it took the poison—1 grain of strychnia; the second died in 37 minutes, and took half the quantity of poison the first did. The first took 5 drachms of tr. camph.; and the second 4 drachms. Was it the extra drachm of camphor that prolonged the life of the first? I think not. J. E. Thompson.

*Bates Co., Mo., August*, 1855.

*Boston Med. and Surg. Journ.*

